# Virtual house calls for Parkinson disease (Connect.Parkinson): study protocol for a randomized, controlled trial

**DOI:** 10.1186/1745-6215-15-465

**Published:** 2014-11-27

**Authors:** Meredith A Achey, Christopher A Beck, Denise B Beran, Cynthia M Boyd, Peter N Schmidt, Allison W Willis, Sara S Riggare, Richard B Simone, Kevin M Biglan, E Ray Dorsey

**Affiliations:** Center for Human Experimental Therapeutics, University of Rochester Medical Center, 265 Crittenden Boulevard, CU420694, Rochester, NY 14642 USA; Department of Biostatistics and Computational Biology, University of Rochester Medical Center, 265 Crittenden Boulevard, Box 630, Rochester, NY 14642 USA; National Parkinson Foundation, 200 SE 1st Street, Suite 800, Miami, FL 33131 USA; Division of Geriatric Medicine and Gerontology, Department of Medicine, Johns Hopkins University School of Medicine, 5200 Eastern Avenue, MFL 7th Floor, Center Tower, Baltimore, MD 21224 USA; Departments of Neurology and of Biostatistics and Epidemiology, Perelman School of Medicine, University of Pennsylvania, 423 Guardian Drive, Office 723, Philadelphia, PA 19104 USA; Department of Learning, Informatics, Management and Ethics, Health Informatics Centre, Karolinska Institute, Tomtebodavägen 18a, Plan 4, S-17177 Stockholm, Sweden; Simone Consulting, 456 Palmetto Drive, Sunnyvale, CA 94086 USA; Department of Neurology, Movement and Inherited Neurological Disorders Unit, University of Rochester Medical Center, 265 Crittenden Boulevard, Box MIND, Rochester, NY 14642 USA

**Keywords:** Health care delivery, Health care disparities, House call, Parkinson disease, Telemedicine, Videoconferencing

## Abstract

**Background:**

Interest in improving care for the growing number of individuals with chronic conditions is rising. However, access to care is limited by distance, disability, and distribution of doctors. Small-scale studies in Parkinson disease, a prototypical chronic condition, have suggested that delivering care using video house calls is feasible, offers similar clinical outcomes to in-person care, and reduces travel burden.

**Methods/Design:**

We are conducting a randomized comparative effectiveness study (Connect.Parkinson) comparing usual care in the community to usual care augmented by virtual house calls with a Parkinson disease specialist. Recruitment is completed centrally using online advertisements and emails and by contacting physicians, support groups, and allied health professionals. Efforts target areas with a high proportion of individuals not receiving care from neurologists. Approximately 200 individuals with Parkinson disease and their care partners will be enrolled at 20 centers throughout the United States and followed for one year. Participants receive educational materials, then are randomized in a 1:1 ratio to continue their usual care (control arm) or usual care and specialty care delivered virtually (intervention arm). Care partners are surveyed about their time and travel burden and their perceived caregiver burden. Participants are evaluated via electronic survey forms and videoconferencing with a blinded independent rater at baseline and at 12 months. All study activities are completed remotely.

The primary outcomes are: (1) feasibility, as measured by the proportion of visits completed, and (2) quality of life, as measured by the 39-item Parkinson’s Disease Questionnaire. Secondary outcomes include measures of clinical benefit, quality of care, time and travel burden, and caregiver burden.

**Discussion:**

Connect.Parkinson will evaluate the feasibility and effectiveness of using technology to deliver care into the homes of individuals with Parkinson disease. The trial may serve as a model for increasing access and delivering patient-centered care at home for individuals with chronic conditions.

**Trial registration:**

This trial was registered on clinicaltrials.gov on January 8, 2014 [NCT02038959].

**Electronic supplementary material:**

The online version of this article (doi:10.1186/1745-6215-15-465) contains supplementary material, which is available to authorized users.

## Background

**Figure 1:**
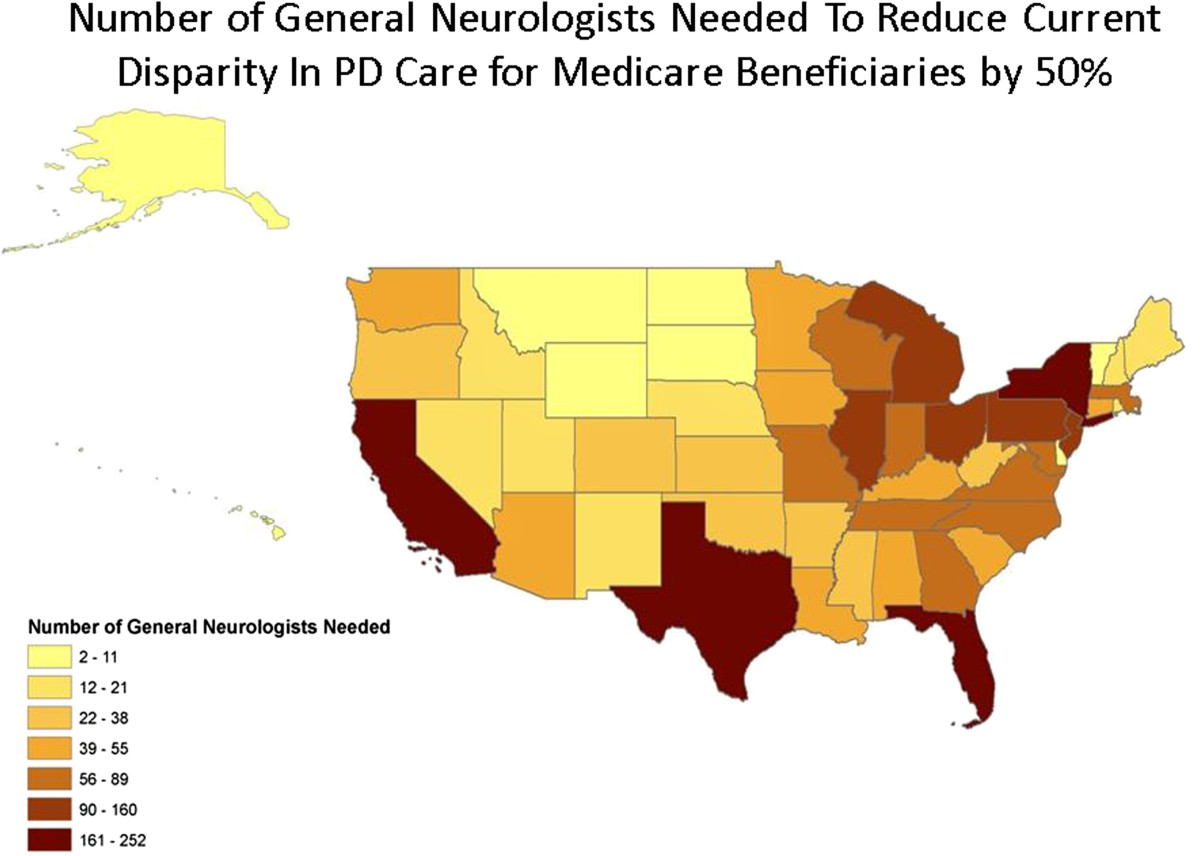
**Number of typical full-time neurologist practices that would need to open to reduce the current disparity in Parkinson disease care by 50%.** Estimates assume that a typical neurologist has 10% of office visits for Parkinson disease patients; that in one year, that neurologist sees patients every six months; and that each neurologist works full-time, five days per week, minus federal holidays and standard vacation.

Chronic conditions affect more than 147 million Americans and account for 85% of U.S. health care expenditures [1]. By 2030, chronic conditions will affect 171 million Americans, or more than half of the U.S. population [1]. Current care for chronic conditions in the United States is costly, ineffectual, often leads to poor outcomes [1–7], and increases burden on caregivers [1, 8, 9]. Many studies have shown that coordinated, multidisciplinary specialty care delivered more frequently can reduce the incidence of acute complications of chronic illness and improve patient and caregiver quality of life [2, 4, 10–14]. Home visits - once a standard mode of care delivery [15] - and care delivered into the home have shown particular promise, especially in caring for older people [16–19]. However, access to such care is frequently limited by distance, disability, and the distribution of specialists (Figure 1), and varies with race and gender [1, 20–22]. Simple, inexpensive videoconferencing technology can alleviate these barriers and provide care to these individuals in their homes.

As videoconferencing technology has become more available, reliable, and less expensive [23–26], interest in using telemedicine to deliver chronic illness care has been increasing [23, 27]. A 2000 Cochrane review of studies comparing telemedicine to face-to-face care concluded, among other things, that ‘Studies of effectiveness, efficiency and appropriateness of telematics applications to health care urgently need to be performed, but technology may permit provision of care which is presently not possible by conventional means’ [27]. Telemedicine interventions including videoconferencing and telemonitoring for veterans with chronic conditions [28], individuals with severe asthma [29], diabetes [30–32], and heart failure [14, 33–43] have all shown promise. In a recent analysis of telemedicine’s applications in chronic disease management, Dr. Richard Wootton reviewed studies of all forms of telemedicine intervention, including remote monitoring and telephone calls, and identified significant problems with the published literature [44]. Studies exploring the use of telemedicine to enable physicians to make virtual house calls have been conducted in a variety of conditions, but have not yet been conducted in Parkinson disease at this scale. We conducted a PubMed search using ‘telemedicine AND home AND randomized’, (336 total results), ‘randomized AND video AND home’ (241 total results), ‘virtual AND visits AND home’ (29 results), and ‘videoconferencing’ AND ‘randomized’ (168 total results), to identify randomized controlled trials reporting on uses of home-based videoconferencing and reviewed the references of Dr. Wootton’s review [44]. Of the 774 search results and 141 studies identified by Dr. Wootton, a total of 16 randomized controlled trials involving remote delivery of care from a physician directly to a patient in the home were identified (Table 1). The present study will be the longest randomized, controlled trial of telemedicine for Parkinson disease of which the authors are aware.

**Table 1:** **Randomized controlled trials involving video-based virtual house calls from physicians**

Study	Year	Sample size	Study population	Intervention(s)	Duration	Primary outcomes	Results
Dorsey ER *et al*. [45]	2013	20	Individuals with Parkinson disease	Randomized to (1) in-person care or (2) care via telemedicine	7 months	• Feasibility	• Virtual house calls were feasible
						• Quality of life	• As effective as in-person care
Fortney JC *et al*. [46]	2013	364	Individuals with depression	Randomized to practice-based or telemedicine-base collaborative care	18 months	• Clinical	• Telemedicine-based collaborative care yielded better outcomes for depressed patients
McCrossan B *et al*. [47]	2012	83	Infants with congenital heart defects	Participants randomized to (1) videoconferencing support, (2) telephone support, or (3) control	10 weeks	• Acceptability	• Clinicians were more confident in treating patients in video visits vs. telephone
						• Health care resource utilization	
							• Parents were satisfied with video visits
							• Health care resource utilization was lower in videoconferencing group
Moreno FA *et al*. [48]	2012	167	Hispanic adults with depression	Randomized to telemedicine care from a psychiatrist or usual care from a primary care physician	6 months	• Clinical	• All participants improved on clinical measures
						• Quality of life	
							• Time to improvement was shorter in telemedicine group
Leon A *et al*. [49] *	2011	83	Individuals with HIV	Randomized to (1) usual care of (2) Virtual Hospital care for one year, then crossed over after one year	2 years	• Clinical	• Satisfaction with Virtual Hospital was high
						• Health care resource utilization	
						• Quality of life	
						• Satisfaction	
							• Clinical outcomes were similar for both groups
Ferrer-Roca O *et al*. [50]	2010	800	Primary care patients referred for specialized care	Randomized to face-to-face hospital referral or telemedicine from specialist	6 months	• Quality of life	• Telemedicine care was comparable to face-to-face care
							• Diagnosis and examination to start treatment were faster in the telemedicine group
Stahl JE, Dixon RF [51]	2010	175	Patients in a general primary care practice	Interviewed face to face and via videoconferencing, order randomized	2 visits	• Satisfaction	• Patients and providers highly satisfied with videoconferencing but preferred face to face
						• Willingness to pay	
							• Technical quality of video calls had significant impact on satisfaction
Dorsey ER *et al*. [52]	2010	14	Individuals with Parkinson disease	Randomized to (1) usual care or (2) care via telemedicine	6 months	• Feasibility	• Virtual house calls were feasible
							• Virtual house calls improved disease-specific measures significantly compared to usual care.
Dixon RF, Stahl JE [53]	2009	175	Patients in a general primary care practice	Randomized to one virtual visit and one face to face visit, or two face to face visits.	2 visits	• Diagnostic agreement	• Physicians and patients highly satisfied with virtual visits
							• Diagnostic agreement between virtual and in-person evaluation was similar to comparison of two in-person evaluations
						• Satisfaction	
Ahmed SN *et al*. [54]	2008	41	Epilepsy patients	Randomized to telemedicine follow-up or conventional	1 visit	• Cost-effectiveness	• 90% of patients in both groups satisfied with quality of services
						• Cost to patients and caregivers	
							• Cost of telemedicine production was similar to patient savings
						• Satisfaction	
Morgan GJ *et al*. [55]	2008	30	Parents of children with severe congenital heart disease	Randomized to telephone or videoconferencing follow-up	6 weeks	• Anxiety	• Videoconferencing decreased anxiety levels compared to telephone and allowed better clinical information
						• Clinical	
O’Reilly R *et al*. [56]	2007	495	Patients referred for psychiatric consult	Randomized to face to face or telepsychiatry	4 months	• Clinical	• Similar outcomes were seen in both arms
						• Cost-effectiveness	
							• Telepsychiatry was at least 10% less expensive than in-person care
						• Satisfaction	
							• Both groups expressed similar satisfaction
De Las Cuevas C *et al*. [57]	2006	140	Psychiatric outpatients	Randomized to face to face or telepsychiatry	24 weeks	• Clinical	• Telepsychiatry had equivalent efficacy to face-to-face care
Ruskin PE *et al*. [58]	2004	119	Veterans with depression	Randomized to telepsychiatry or in-person psychiatrist visits	6 months	• Clinical	• Both groups were equivalent in clinical outcomes, cost, patient adherence, and patient satisfaction.
						• Cost-effectiveness	
						• Health care resource utilization	
						• Satisfaction	
Bishop JE *et al*. [59]	2002	19	Psychiatric patients	Randomized to videoconference or face to face	4 months	• Satisfaction	• Similar satisfaction observed in both groups

^*^Study evaluates an intervention that includes virtual house calls, but also includes other telemonitoring or electronic communication methodologies.

Parkinson disease is a prototypical chronic condition in which to test this health care delivery method. Like many chronic conditions, Parkinson disease has an incidence that increases with age [60], a long duration (average survival of approximately 14 years after diagnosis) that results in progressive disability [61], impairs driving ability [62, 63], burdens caregivers [64], often requires institutional care [61, 65–68], generates high health care costs to private and public payers [69], and, importantly, benefits from specialized care [12, 21, 22, 70]. However, over 40% of Medicare beneficiaries with Parkinson disease do not receive neurologic care within four years of diagnosis, and those who have not are at a yearly 14% increased risk of hip fracture, 21% increased risk of placement in skilled nursing facilities within the first year, and a 22% increased risk of death within six years compared to those who see a neurologist [21, 22]. Because many of its symptoms can be readily assessed visually, interest in using telemedicine to facilitate care began over 20 years ago [71] and has increased to the present [72]. Pilot studies using web-based videoconferencing have previously shown the efficacy, value, and acceptability of virtual house calls from specialists to people with Parkinson disease [45, 52, 73]. Virtual house calls can also incorporate multidisciplinary care and education from a team of health care providers, which has been shown to be highly effective for Parkinson disease [10, 11, 74–79]. The present study will add to understanding of the promise and limitations of virtual house calls for the treatment of Parkinson disease.

The trial was registered on clinicaltrials.gov in January 2014 (NCT02038959).

## Methods

### Trial design

We are conducting a randomized comparative effectiveness study (Connect.Parkinson) comparing usual care in the community to usual care augmented by video house calls with a Parkinson disease specialist [80]. Approximately 200 individuals with Parkinson disease and their care partners will be enrolled at 20 centers throughout the United States and followed for one year. Participants receive educational materials, then are randomized in a 1:1 ratio to continue their usual care (control arm) or usual care and specialty care delivered virtually (intervention arm). Care partners are surveyed about their time and travel burden and their perceived caregiver burden. A blinded independent rater and a study coordinator conduct baseline and end-of-study assessments. All study activities are completed remotely. The specific aims of the study are: (1) to demonstrate the feasibility of using virtual house calls to deliver specialty care into the homes of individuals with Parkinson disease who have limited access to care; (2) to show that such an approach can improve quality of life; (3) to establish that virtual house calls can enhance the quality of care; and (4) to demonstrate that this remote approach to care saves time, reduces travel, and decreases care partner burden.

To conduct the study, we have partnered with the largest Parkinson disease patient organization in the country, the National Parkinson Foundation, and formed a Patient Advisory Board with patients and patient advocates who have contributed to the design of the trial and continue to be involved with the project. Finally, we have assembled a Dissemination and Implementation Advisory Board to assist in disseminating the results of the research and drive broader adoption.

This study was approved by the Research Subjects Review Board of the University of Rochester as a coordinating center (January 2014) and an enrolling site (March 2014). As of October 26, 2014, the study has been approved at 16 sites and is under review at four additional sites (Additional file 1).

### Participants

Eligibility criteria were designed to permit broad participation in the study. Individuals with clinically diagnosed idiopathic Parkinson disease, who have access to a non-public, internet-enabled device with the capacity for videoconferencing, who are physically located in a state where a participating site investigator is licensed to practice medicine when visits are conducted, and are willing and able to provide informed consent, may enroll. Participants must also have a local health care provider (for example primary care physician, nurse practitioner) who the study team can contact to provide recommendations from the site investigators, and must live at home, in a senior housing complex or assisted living facility. Individuals who are currently hospitalized, enrolled in another telemedicine study, or who have a condition (for example, prominent psychosis) that precludes study participation will be excluded from study participation.

Care partners must be adults who are able and willing to provide informed consent to be enrolled. Their participation is optional.

### Procedures

Individuals with Parkinson disease will be recruited and enrolled remotely and sent educational materials about Parkinson disease created by the National Parkinson Foundation. They will also be asked to identify their regular care partners (friends or family members who provide regular assistance with daily activities and are not paid caregivers), who will be invited to enroll. Enrollment is completed in two parts; first, a central study coordinator reviews an interest survey and contacts interested individuals to verify their eligibility and complete a screening form, then enrolling site staff contacts the potential participant to obtain consent. Consent is obtained with a written signature on a printed consent form. All study activities are completed remotely, using email, phone, fax, mail, and videoconferencing modalities to enable individuals to participate from home. Study data are collected and managed using Research Electronic Data Capture (REDCap) tools hosted at the University of Rochester [81]. REDCap is a secure, web-based application designed to support data capture for research studies, providing an interface for validated data entry; audit trails for tracking data manipulation and export procedures, automated export procedures for seamless data downloads to common statistical packages; and procedures for importing data from external sources if needed. REDCap supports the use of electronic patient surveys and automated email invitations, which are used in this study to allow participant-completed assessments to be done securely from home, with the aid of a family member if needed. A complete schedule of activities is included in Additional file 2.

Participants who enroll will be emailed a link to download secure Health Insurance Portability and Accountability Act-compliant virtual visit software from SBR Health (Cambridge, MA, USA). The software embeds videoconferencing software from Vidyo (Hackensack, NJ, USA) that is hosted by ID Solutions (Indianapolis, IN, USA), which uses two-way encrypted video transmission to ensure privacy. SBR Health also creates a virtual waiting room that allows patients to ‘check in’ for appointments. If participants do not have access to a webcam, a Creative Labs Live! Cam Chat HD camera is mailed to them prior to their baseline assessment virtual visit. A study coordinator at the University of Rochester performs a test connection with the participants, providing technical support by phone if needed. No in-person technical support is sent to the participant’s home.

Participants will be evaluated via videoconferencing and via electronically administered surveys at baseline and at 12 months. Blinded independent raters complete remote baseline and end-of-study (12-month) assessments of Parkinson disease using the Movement Disorder Society Unified Parkinson Disease Rating Scale (MDS-UPDRS) [82] modified (excludes assessment of tone and balance) for remote assessment [83]. Individuals who the independent rater believes not to have Parkinson disease are withdrawn at the baseline visit prior to randomization. A study team member also completes a remote Montreal Cognitive Assessment (MoCA) [84, 85] at this visit. Additional baseline assessments are completed by the participant/care partner and study staff as detailed in Additional file 2. Care partners are surveyed at baseline and at the end of the study about the time and travel required to help the participant with their Parkinson disease appointments, and the perceived burden of caring for the individual with Parkinson disease. All participant-completed study assessments are completed via secure survey links sent to their email addresses using REDCap, and study teams enter data from each visit directly into the study database.

### Randomization

After the initial evaluation, participants are randomized to either continue with their usual care throughout the year or to continue their usual care and receive virtual visits from a Parkinson disease specialist licensed to practice in the state in which they reside. The randomization allocation sequence was generated by C.A.B. using R version 3.0.2. Randomization is conducted in the study’s REDCap database after the baseline assessments have been completed and Parkinson disease diagnosis confirmed. The randomization plan is stratified by enrolling site and contains blocking to ensure approximately even distribution of control/treatment arm participants.

### Interventions

Participants with Parkinson disease are randomly assigned to either continue with their usual care or continue with usual care supplemented by virtual house calls. The care received by the usual care (control) group will be variable but will be a reflection of the status quo for Parkinson disease care in the United States. This group is free to seek out specialty care over the course of the study, and we anticipate that some may do so. Those assigned to usual care are given the opportunity to have a one-time virtual visit with a Parkinson disease specialist after their final study assessment. For the telemedicine (intervention) group, the visit schedule is set by the investigator in consultation with the patient and will include at least four virtual visits over one year. Visits are similar to regular in-person clinical visits for Parkinson disease, and investigators provide a clinical note summarizing the visit and any recommendations for treatment to the patients and their local health care providers at the conclusion of each visit.

### Outcome measures

Primary outcomes include (1) feasibility, defined as the percentage of telemedicine participants who complete at least one telemedicine visit, and the overall percentage of completed telemedicine visits, and (2) quality of life, measured by the change in the 39-item Parkinson’s Disease Questionnaire (PDQ-39) [86] from baseline to 12 months.

Secondary outcomes include quality of care, as measured by the change from baseline in the Patient Assessment of Chronic Illness Care (PACIC) [87], and time and travel savings from remote appointments, and change in caregiver burden as measured by the Multidimensional Caregiver Strain Index (MCSI) [88]. Additional secondary outcomes have been selected to determine the impact of telemedicine specialist care on Parkinson disease-specific outcomes and global quality of life. The change in Parkinson disease symptoms and signs will be assessed by the change in the MDS-UPDRS from baseline to 12 months. In addition, changes in depression and cognition, common comorbidities with Parkinson disease [89–92], will be identified as the change in the 15-item Geriatric Depression Scale (GDS-15) [93] and the MoCA. Additional quality-of-life metrics are the Patient Global Impression of Change [94] and the European Quality of Life Five Dimension Five Level Scale (EuroQoL-5D-5 L) [95]. Patient-reported utilization of health care services such as hospitalizations, emergency room visits, and visits to primary care doctors [96] will also be compared between the control and intervention arms.

### Planned statistical analyses

The aims of the study are to evaluate the feasibility, quality of life, clinical benefit, quality of care, and value of using telemedicine to deliver specialty care to patients in their home. Primary measures of feasibility will be summarized using descriptive statistics. We will consider telemedicine to be feasible if 80% of participants in the telemedicine arm complete at least one telemedicine visit, and at least 80% of all telemedicine visits are completed as scheduled. Generalized linear mixed models will be used to determine what factors affect the probability of completing telemedicine visits as scheduled.

The primary efficacy outcome measure of this study is the PDQ-39. For this outcome, we will fit an analysis of covariance model with the change in PDQ-39 from baseline to one year as the response, treatment group as the factor of interest, participating physician as a stratification factor, and baseline PDQ-39 as a covariate. A *t* test will be performed to compare the adjusted treatment group means. Secondary measures of quality of life, clinical benefit, quality of care, and value to patients and care partners will be analyzed similarly. Additional analyses will examine the relationships among outcome variables. All statistical tests will be performed at the two-sided significance level of 5%, and no corrections will be made for multiple testing.

### Sample size

The sample size of 200 Parkinson disease patients was selected to ensure adequate power (80 to 90%) to detect a moderate effect size on the PDQ-39 (Cohen’s d of 0.5) using a two-sided *t* test at a significance level of 5% allowing for an anticipated dropout rate of up to 20%.

### Recruitment

Recruitment for the study began in February 2014. Recruitment methods were designed to reach the large number of patients with Parkinson disease who do not currently see a neurologist. To address disparities in access to care, we have identified and continue to target underserved areas nationally, defining counties as ‘underserved’ as those in which a majority of Medicare beneficiaries diagnosed with Parkinson disease have not seen a neurologist [20]. We have created targeted Google AdWords to display for searches related to Parkinson disease in these defined geographies. We have also identified primary care providers who may see a large proportion of Parkinson disease patients and will send study materials to these practices in eligible states to recruit patients from these areas. We have built a website (Connect.Parkinson.org) based on the study flier and created an informational video featuring a member of our Patient Advisory Board. Interested individuals contact the National Parkinson Foundation PD Helpline (800.4PD.INFO) for information about the study and can submit their contact information to the coordinating center through the Helpline or directly through a survey form on the website. Additional methods of recruitment include outreach to support groups and trained allied health professionals (for example, physical therapists) in underserved areas. We supplement these efforts by announcing the study through communications to the National Parkinson Foundation’s distribution list, a Clinical Trial Announcement through the patient social networking site PatientsLikeMe, and by posting the study in online patient communities such as the Michael J. Fox Foundation’s Fox Trial Finder. Based on our objective to reach those with limited access to care, we will prioritize enrollment of individuals who are not seeing a neurologist or come from an underserved region.

## Discussion

Telemedicine holds tremendous promise for increasing access and quality of care and decreasing cost for chronic conditions. Video visits into the home represent a new generation of house calls, poised to bring about the return of this personalized, convenient, and accessible care model [18]. The Connect.Parkinson study aims to demonstrate the feasibility and efficacy of using home telemedicine for individuals with Parkinson disease. This effort is one of the largest and longest randomized controlled trials assessing this care delivery model for a chronic condition and will involve providers and patients with little previous experience of telemedicine. Large-scale implementation of this method of care will depend in part on physician and patient adoption of this care model [97, 98]. Even though the means of communication used in this study are common in everyday life (for example, grandparents use videoconferencing to connect to their grandchildren), the use of this technology to deliver care may still appear foreign to many.

**Figure 2:**
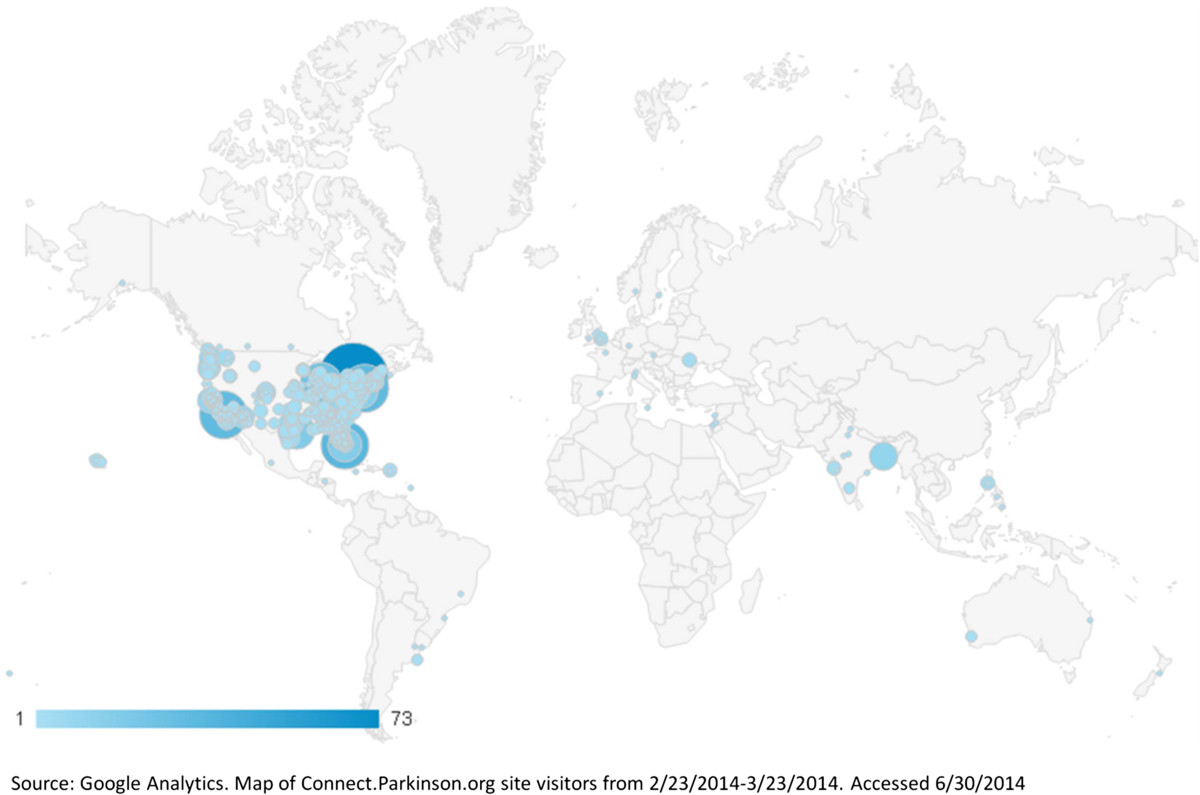
**Individuals from all over the world have accessed the Connect.Parkinson study website at** **.** http://connect.parkinson.org

**Figure 3:**
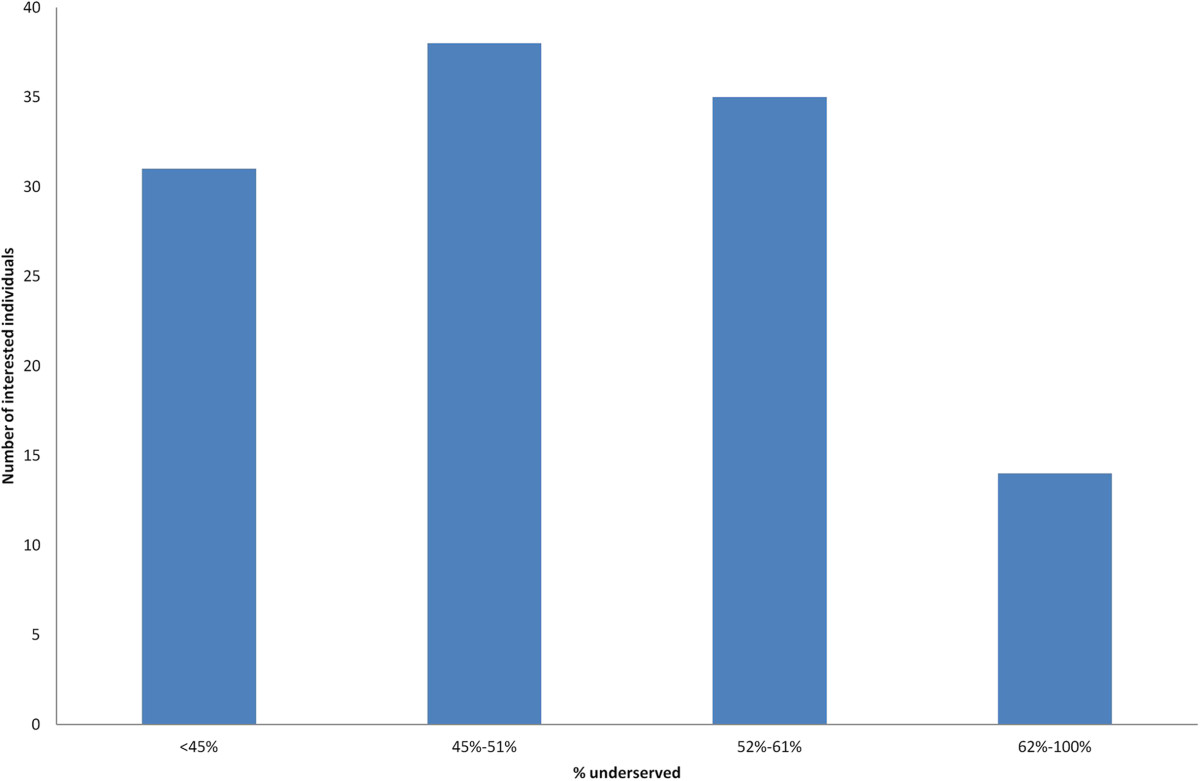
**Potential Connect.Parkinson participants in underserved zip codes.** Distribution of interested individuals by the proportion of underserved patients with Parkinson disease in their zip code. Data current as of May 20, 2014.

Interest in the study has been robust. In the first month (February 2014 to March 2014) in response to limited outreach, over 1,400 individuals visited the Connect.Parkinson website from all over the United States and the world (Figure 2) and over 300 completed an online survey expressing interest in participating in the study. Efforts to reach those with limited access to care have been more challenging. Most interested individuals in the first four months of recruiting efforts came from individuals in underserved areas (Figure 3); however, most of the respondents are seeing a neurologist regularly, suggesting that time and travel burden may be driving interest. Considering the known differences between the demographics of clinical trial participants and those of the general population and Medicare beneficiaries in particular, these data are not surprising [99, 100]. We will continue efforts to facilitate inclusion of individuals who may be having difficulty accessing neurologists.

The study has additional limitations related to the availability of the technology and the nature of the visits. While broadband access is increasingly common [101, 102], a digital divide still exists [25]. In particular, individuals with chronic medical conditions report significantly less internet access than those without [103]. This divide may indeed limit our ability to connect to many of the patients in underserved areas who we are trying to reach. Even for those who are able to connect, the quality of connection is often highly variable and dependent on the speed of the patient’s internet connection. This issue may be more prominent in older people. While 86% of American adults use the internet, only 59% of adults over 65 do so, and only 47% have a broadband internet connection at home [104]. Only 15% of adults over 65 reported using videoconferencing in 2010 [105], and the technology is still foreign to many, especially when applied to health care. Even those who do have internet connectivity may be using older hardware and operating systems that do not readily support the newer videoconferencing software, or may have limited familiarity with installing software, both of which can cause delays in setting up and conducting visits and interfere with the quality of the assessments. These delays and the participants’ baseline familiarity with the internet and related technologies are being measured as part of this study. The necessity of obtaining written signatures for consent forms (in lieu of electronic signatures) also introduces a delay in the enrollment process. Signed consent forms must be sent to sites via mail or email, creating unnecessary delays. This has been the case with other primarily internet-based clinical trials [106]. With the continuing integration of internet-based communications into all aspects of medicine and research, methods of obtaining electronic signatures securely should increasingly become part of standard research practice, as they have been implemented successfully in other primarily internet-based clinical trials [107–109].

In addition to the study’s technological limitations, the nature of the remote visits is limited. While several studies have demonstrated that the standard Parkinson disease rating scale can be administered remotely and that remote assessments closely correlate with in-person assessments [83, 110–112], the quality of the examination is not as good as in person. As such, assessments of tone (for example, for cogwheel rigidity) and balance (for example, a ‘pull’ test in which patients are pulled backward by an examiner) are not feasible. Similarly, assessments of more subtle signs, such as eye movements, can be more difficult remotely. Notwithstanding these limitations, it should be noted that the seminal description of the disease nearly two centuries ago by Dr. James Parkinson in 1817 including the cardinal features of rest tremor, bradykinesia (slowness in movement), and gait imbalance was based almost exclusively on his visual observations of individuals walking in a London park [113]. Beyond the technical assessment, the personal connection between a patient and physician is limited by the absence of physical touch. However, studies of telemedicine have largely found the quality of the interpersonal connection between patients and physicians to be high [52, 74, 114] and patients with Parkinson disease experiencing virtual visits for the first time have highlighted care (including access to specialists), convenience (absence of travel), and comfort (including privacy) as benefits of telemedicine [73], suggesting that remote visits are qualitatively different and not necessarily inferior to in-person visits.

Broader adoption of telemedicine is also limited by regulatory and reimbursement barriers. Currently, most state licensing boards require that physicians be licensed in the state where the patient is physically located when services are provided [115]. Consequently, many patients often cannot access care from specialists simply because of where they live. The state of Delaware, for example, has no Parkinson disease specialists, leaving patients who desire such care having to drive hours to major urban centers (for example, Baltimore or Philadelphia). In addition, payers have been slow to reimburse for telemedicine. While an increasing number of states mandate that private insurers cover telemedicine to the extent they cover in-person care [116], many of these mandates do not extend to care in the home. In addition, Medicare does not cover care provided virtually in the home. In fact, Medicare pays more for care provided in high-cost, often patient-unfriendly, institutions (for example, hospitals) than it does for care in the community [117]. Where such licensing and reimbursement barriers do not exist (for example, Canada [118, 119], prisons [120–125], and the Department of Veterans Affairs [28, 126]) telemedicine in its various forms, including care into the home, has flourished.

The Connect.Parkinson study aims to contribute valuable information about the feasibility, effectiveness, and value of using technology to deliver care to patients with Parkinson disease directly in their home. The dissemination of the results, aided by the Dissemination and Implementation Advisory Board, will help break down many of the barriers to adoption of this care model and bring us closer to enabling anyone anywhere to receive care.

## Trial status

Currently recruiting participants.

## Electronic supplementary material

Additional file 1: Connect.Parkinson participating sites.(DOC 44 KB)

Additional file 2: Schedule of activities.(DOC 46 KB)

Below are the links to the authors’ original submitted files for images.

Authors’ original file for figure 1

Authors’ original file for figure 2

Authors’ original file for figure 3

## Abbreviations

EQ-5D-5 L/EuroQoL-5D-5 L: European Quality of Life Five Dimension Five Level Scale
GDS-15: 15-item Geriatric Depression Scale
MCSI: Multidimensional Caregiver Strain Index
MDS-UPDRS: Movement Disorders Society Unified Parkinson Disease Rating Scale
MoCA: Montreal Cognitive Assessment
PACIC: Patient Assessment of Chronic Illness Care
PDQ-39: 39-item Parkinson’s Disease Questionnaire
REDCap: Research Electronic Data Capture.
