# Erratum To: Virtual house calls for Parkinson disease (Connect.Parkinson): study protocol for a randomized, controlled trial

**DOI:** 10.1186/s13063-015-0984-7

**Published:** 2016-01-05

**Authors:** Meredith A. Achey, Christopher A. Beck, Denise B. Beran, Cynthia M. Boyd, Peter N. Schmidt, Allison W. Willis, Sara S. Riggare, Richard B. Simone, Kevin M. Biglan, E. Ray Dorsey

**Affiliations:** Division of Geriatric Medicine and Gerontology, Department of Medicine, Johns Hopkins University School of Medicine, 5200 Eastern Avenue, MFL 7th Floor, Center Tower, Baltimore, MD 21224 USA

After the publication of this article [1], it was discovered that eleven of the trials listed in the original article's Table 1 [1], had been erroneously identified as taking place in the home [2–12]. These studies actually evaluated physician videoconferencing visits with patients located in clinics. To ensure accuracy, we repeated the literature search in September of 2015, using the same search terms reported in the article and filtered for a publication date prior to July 1, 2014 (the original work was performed in June 2014.) We searched PubMed using the terms ‘telemedicine AND home AND randomized’ (378 results), ‘randomized AND video AND home’ (259 results), ‘videoconferencing AND randomized’ (178 results), and ‘virtual AND visits AND home’ (33 results), and reviewed the 141 studies identified in the review by Dr. Wootton mentioned in the article [13]. Of the 848 search results and 141 studies identified by Dr. Wootton, a total of six randomized controlled trials involving physician video calls directly to a patient in the home were identified (four from the original review [14–17] and two additional studies [18, 19] identified through the new search). The eleven misidentified articles have been removed from the Corrected Table 1, and included for clarity as Erratum Table 2. The final paper listed in Erratum Table 2, Bishop JE et al. [3], has also been corrected here: our article reported 19 subjects, but the abstract indicates that 17 completed the study. We sincerely apologize for the oversight and any inconvenience these errors might have caused.

**Corrected Table 1:** Randomized, controlled trials involving video based virtual house calls from physicians (N = 6)

Study	Year	Sample size	Study population	Intervention(s)	Duration	Primary outcomes	Results
Dorsey ER et al. [14]	2013	20	Individuals with Parkinson disease	Randomized to (1) in-person care or (2) care via telemedicine	7 months	• Feasibility	• Virtual house calls were feasible
						• Quality of life	• As effective as in-person care
McCrossan B et al. [15]	2012	83	Infants with congenital heart defects	Randomized to (1) videoconferencing support, (2) telephone support, or (3) control	10 weeks	• Acceptability	• Clinicians were more confident in treating patients in video visits vs. telephone
						• Healthcare resource utilization	• Parents were satisfied with video visits • Healthcare resource utilization was lower in video-conferencing group
Leon A et al. [17]^a^	2011	83	Individuals with HIV	Randomized to (1) usual care or (2) Virtual Hospital care for one year, then crossed over after one year	2 years	• Clinical	• Satisfaction with Virtual Hospital was high
						• Healthcare resource utilization	• Clinical outcomes were similar for both groups
						• Quality of life	
						• Satisfaction	
Morgan GJ et al. [16]	2008	30	Parents of children with severe congenital heart disease	Randomized to (1) telephone or (2) videoconferencing follow-up	6 weeks	• Parents’ anxiety	• Videoconferencing decreased anxiety levels compared to telephone and allowed better clinical information
						• Clinical	
						• Clinician and patient satisfaction	
Dallolio L et al. [19]	2008	137	Individuals with spinal cord injury	Randomized to (1) home (or nursing home or hospital) telemedicine (physician and nurse) and telerehabilitation (therapist) or (2) standard post-discharge care	6 months	• Clinical	• Telemedicine patients at one out of four sites had statistically significantly better functional improvement
						• Satisfaction	• Satisfaction with interactions with nursing and medical staff and information and treatment received were higher in the telemedicine group
Whitlock WL et al. [18]^a^	2000	28	Individuals with Type II diabetes	Randomized to (1) home videoconferencing (monthly physician calls and weekly nurse calls) or (2) standard in-person care	3 months	• Clinical	• Some clinical outcomes improved significantly more in the telemedicine group
						• Quality of life	• Quality of life was unchanged
						• Satisfaction	• Physicians and case managers reported high subjective utility of telemedicine
							• Technology problems were an obstacle

^a^Study evaluates an intervention that includes virtual house calls, but also includes other telemonitoring and/or electronic communication methodologies

**Erratum Table 2:** Randomized, controlled trials involving video based physician visits with patients in clinical environments (N = 11)

Study	Year	Sample size	Study population	Intervention(s)	Duration	Primary outcomes	Results
Fortney JC et al. [8]	2013	364	Individuals with depression	Randomized to practice-based or telemedicine-base collaborative care	18 months	• Clinical	• Telemedicine-based collaborative care yielded better outcomes for depressed patients
Moreno FA et al. [9]	2012	167	Hispanic adults with depression	Randomized to telemedicine care from a psychiatrist or usual care from a primary care physician	6 months	• Clinical	• All participants improved on clinical measures
						• Quality of life	• Time to improvement was shorter in telemedicine group
Ferrer-Roca O et al. [7]	2010	800	Primary care patients referred for specialized care	Randomized to face-to-face hospital referral or telemedicine from specialist	6 months	• Quality of life	• Telemedicine care was comparable to face-to-face care
							• Diagnosis and examination to start treatment were faster in the telemedicine group
Stahl JE, Dixon RF [12]	2010	175	Patients in a general primary care practice	Interviewed face-to-face and via videoconferencing, order randomized	2 visits	• Satisfaction	• Patients and providers were highly satisfied with videoconferencing but preferred face-to-face
						• Willingness to pay	• Technical quality of video calls had significant impact on satisfaction
Dorsey ER et al. [6]	2010	14	Individuals with Parkinson disease	Randomized to usual care or care via telemedicine	6 months	• Feasibility	• Virtual house calls were feasible
							• Virtual house calls improved disease-specific measures significantly compared to usual care.
Dixon RF, Stahl JE [5]	2009	175	Patients in a general primary care practice	Randomized to one virtual visit and one face-to-face, or two face-to-face consultations	2 visits	• Diagnostic agreement	• Physicians and patients highly satisfied with virtual visits
						• Satisfaction	• Diagnostic agreement between virtual and in-person evaluation was similar to comparison of two in-person evaluations
Ahmed SN et al. [2]	2008	41	Epilepsy patients	Randomized to telemedicine follow up or conventional	1 visit	• Cost effectiveness	• 90 % of patients in both groups satisfied with quality of services
						• Cost to patients and caregivers	• Cost of telemedicine production was similar to patient savings
						• Satisfaction	
O’Reilly R et al. [10]	2007	495	Patients referred for psychiatric consult	Randomized to face to face or telepsychiatry	4 months	• Clinical	• Similar outcomes were seen in both arms
						• Cost effectiveness	• Telepsychiatry was at least 10 % less expensive than in-person care
						• Satisfaction	• Both groups expressed similar satisfaction
De Las Cuevas C et al. [4]	2006	140	Psychiatric outpatients	Randomized to face-to-face or telepsychiatry	24 weeks	• Clinical	• Telepsychiatry had equivalent efficacy to face-to-face care
Ruskin PE et al. [11]	2004	119	Veterans with depression	Randomized to telepsychiatry or in-person psychiatrist visits	6 months	• Clinical	• Both groups were equivalent in clinical outcomes, cost, patient adherence, and patient satisfaction.
						• Cost effectiveness	
						• Healthcare resource utilization	
						• Satisfaction	
Bishop JE et al. [3]	2002	17	Psychiatric patients	Randomized to videoconference or face-to-face	4 months	• Satisfaction	• Similar satisfaction observed in both groups
