# Supplementary material for: Virtual house calls for Parkinson disease (Connect.Parkinson): study protocol for a randomized, controlled trial
Source: Trials. 2014 Nov 27;15:465. doi: 10.1186/1745-6215-15-465 (PMC4289172; doi:10.1186/1745-6215-15-465)
Supplement: Supplementary file 1 — Additional file 1: Connect.Parkinson participating sites.(DOC 44 KB) [file 13063_2014_2358_MOESM1_ESM.doc]

**Additional file 1: Connect.Parkinson Participating Sites**

| **Site Name** | **IRB approval date of Protocol** | **IRB Name** | **IRB Number Assigned** |
| --- | --- | --- | --- |
| University of Rochester (Coordinating Center) | 1/6/2014 | Research Subjects Review Board | RSRB #49982 |
| University of Rochester (Enrolling Site) | 3/21/2014 | Research Subjects Review Board | RSRB #50757 |
| Oregon Health and Science University | 5/27/2014 | OHSU IRB | IRB00010694 |
| Northwest Neurological, PLLC | 5/29/2014 | Western IRB | 1147023 |
| Northwestern | 5/29/2014 | Northwestern University Biomedical IRB | STU00093785 |
| Beth Israel Deaconess | 6/16/2014 | Committee on Clinical Investigations | 2014-P-000130/1 |
| Duke Medical Center | 6/17/2014 | Duke University Health System Institutional Review Board for Clinical Investigations | Pro00055073 |
| University of Kansas Medical Center | 7/21/2014 | KU Medical Center Institutional Review Board | STUDY00001308 |
| University of Pennsylvania | 7/21/2014 | University of Pennsylvania IRB | 820520 |
| Georgia Regents University | 7/24/2014 | IRBNet | [625518-1] |
| Johns Hopkins | 8/3/2014 | Johns Hopkins Medicine IRB-X | IRB00034103 |
| Mayo Clinic | 8/25/2014 | Mayo Clinic IRB | 14-002259 |
| Massachusetts General Hospital | 9/19/2014 | Partners Human Research Committee | 2014P001038/MGH |
| Struthers | 9/23/2014 | Park Nicollet Institute IRB | 04368-14-A |
| University of California San Francisco | 9/29/2014 | UCSF Committee on Human Research (IRB #14-13290) | 087114 |
| Baylor College of Medicine | 10/2/2014 | BCM IRB | H-34712 |
| North Shore Long Island Jewish | 10/3/2014 | North Shore-Long Island Jewish Health System IRB | 14-272B |
| Parkinson's Institute | under review |  |  |
| University of Florida | under review |  |  |
| University of Miami | under review |  |  |
| Medical University of South Carolina | under review |  |  |
|  |  |  |  |
| **List current as of:** | 10/26/2014 |  |  |
