# Supplementary material for: Virtual house calls for Parkinson disease (Connect.Parkinson): study protocol for a randomized, controlled trial
Source: Trials. 2014 Nov 27;15:465. doi: 10.1186/1745-6215-15-465 (PMC4289172; doi:10.1186/1745-6215-15-465)
Supplement: Supplementary file 2 — Additional file 2: Schedule of activities.(DOC 46 KB) [file 13063_2014_2358_MOESM2_ESM.doc]

| **Additional file 2: Schedule of Activities** | | | | | | | | | |
| --- | --- | --- | --- | --- | --- | --- | --- | --- | --- |
| **Activity** | **Screening**  **visit**  **(phone)** | **Consent**  **(phone or in person)** | **Baseline assessment**  **(video call and survey)** | **Virtual visits 1 and 4**  **(months 0 and 12)** | | **Virtual visits 2 and 3** | **End of study assessment**  **(video call and survey)** | **One-time Virtual Visit**  **(video call)** | **End of Study** |
| ***Study Personnel*** | *Study Coordinator* | *Site Coordinator* | *Independent rater* | *Site Investigator* | | *Site Investigator* | *Independent rater* | *Site Investigator* | *Site Investigator* |
| **Study Site** | **Rochester** | **Site** | **Rochester** | **Site** | | **Site** | **Rochester** | **Site** | **Site** |
| Participant Arm | All | All | All | Telemedicine | | Telemedicine | All | Control | N/A |
| Screening | X |  |  |  | |  |  |  |  |
| Contact local health care provider | X |  |  |  | |  |  |  |  |
| Consent |  | X |  |  | |  |  |  |  |
| Baseline Survey |  |  | X |  | |  |  |  |  |
| Randomization |  |  | X |  | |  |  |  |  |
| Assessments of Health, Quality of Life, and Care* |  |  | X |  | |  | X |  |  |
| EuroQoL-5D-5L |  |  | X |  | |  | X |  |  |
| Medication log |  |  | X |  | |  | X |  |  |
| MoCA |  |  | X |  | |  | X |  |  |
| MDS-UPDRS |  |  | X |  | |  | X |  |  |
| PGIC |  |  |  |  | |  | X |  |  |
| Time and Travel + MCSI** |  |  | X |  | |  | X |  |  |
| Virtual Visit survey*** |  |  |  | X | | X |  | X |  |
| Overall Virtual Visit survey |  |  |  |  | |  | X | X |  |
| Provider Virtual Visit survey**** |  |  |  |  | |  |  |  | X |
| MDS-UPDRS Part III**** |  |  |  | X | |  |  |  |  |
| Clinic note sent to participant and local health care provider |  |  |  | X | | X |  |  |  |
| * Includes Parkinson’s Disease Questionnaire 39, Geriatric Depression Scale 15, Patient Assessment of Chronic Illness Care, Time and travel, Parkinson disease recommendation survey, and healthcare resource utilization.  Abbreviations: MoCA = Montreal Cognitive Assessment; MDS-UPDRS = Movement Disorder Society Unified Parkinson Disease Rating Scale; PGIC = Patient Global Impression of Change; MCSI = Multidimensional Caregiver Strain Index | | | | | | | | | |
| **For participating care partners  *** Participants and investigators | | | | | **** For investigators giving care via telemedicine | | | | |
