# Supplementary figures and images for: Virtual house calls for Parkinson disease (Connect.Parkinson): study protocol for a randomized, controlled trial
Source: Trials. 2014 Nov 27;15:465. doi: 10.1186/1745-6215-15-465 (PMC4289172; doi:10.1186/1745-6215-15-465)

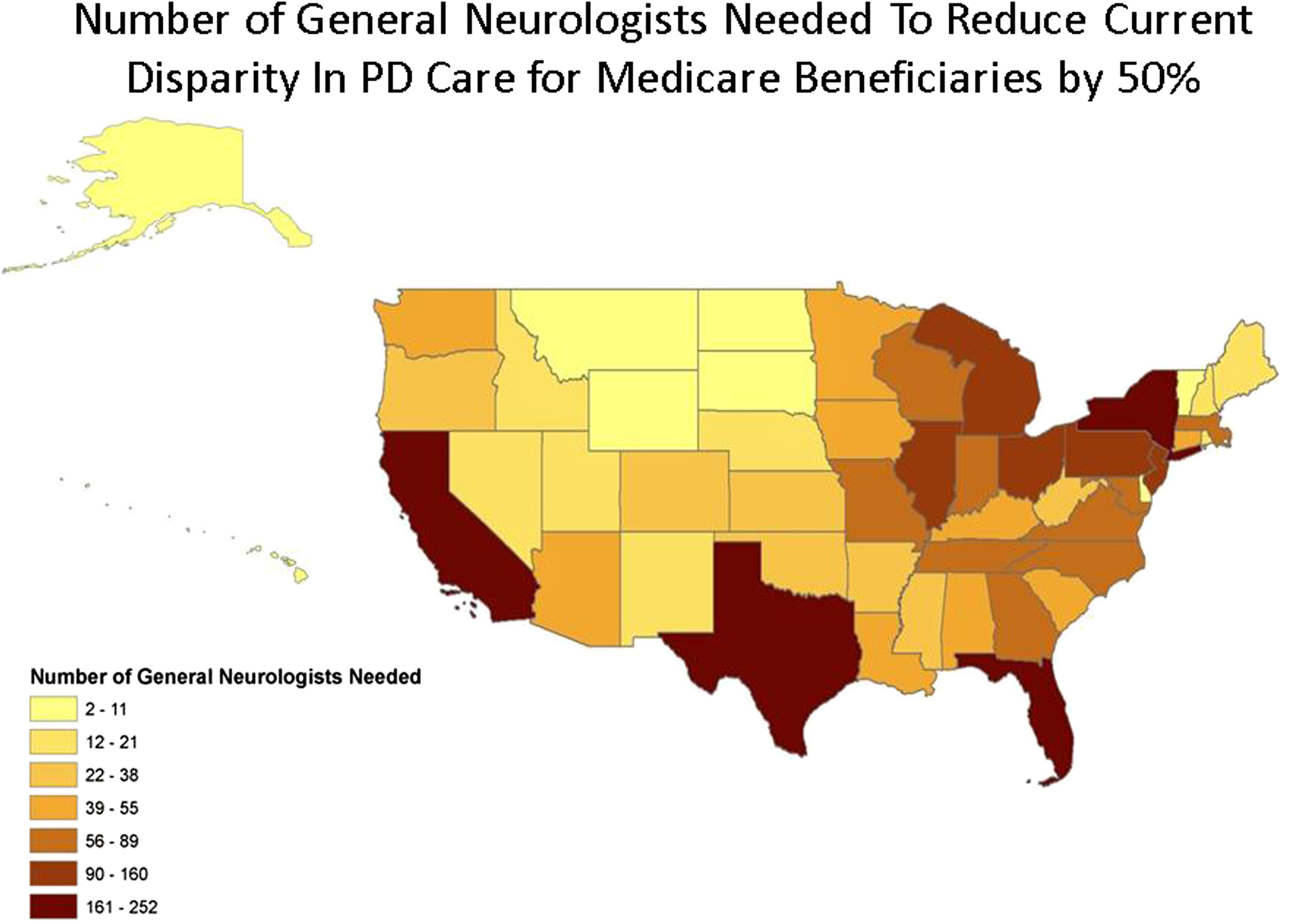

Supplement: Supplementary file 3 — Authors’ original file for figure 1 [file 13063_2014_2358_MOESM3_ESM.tif]

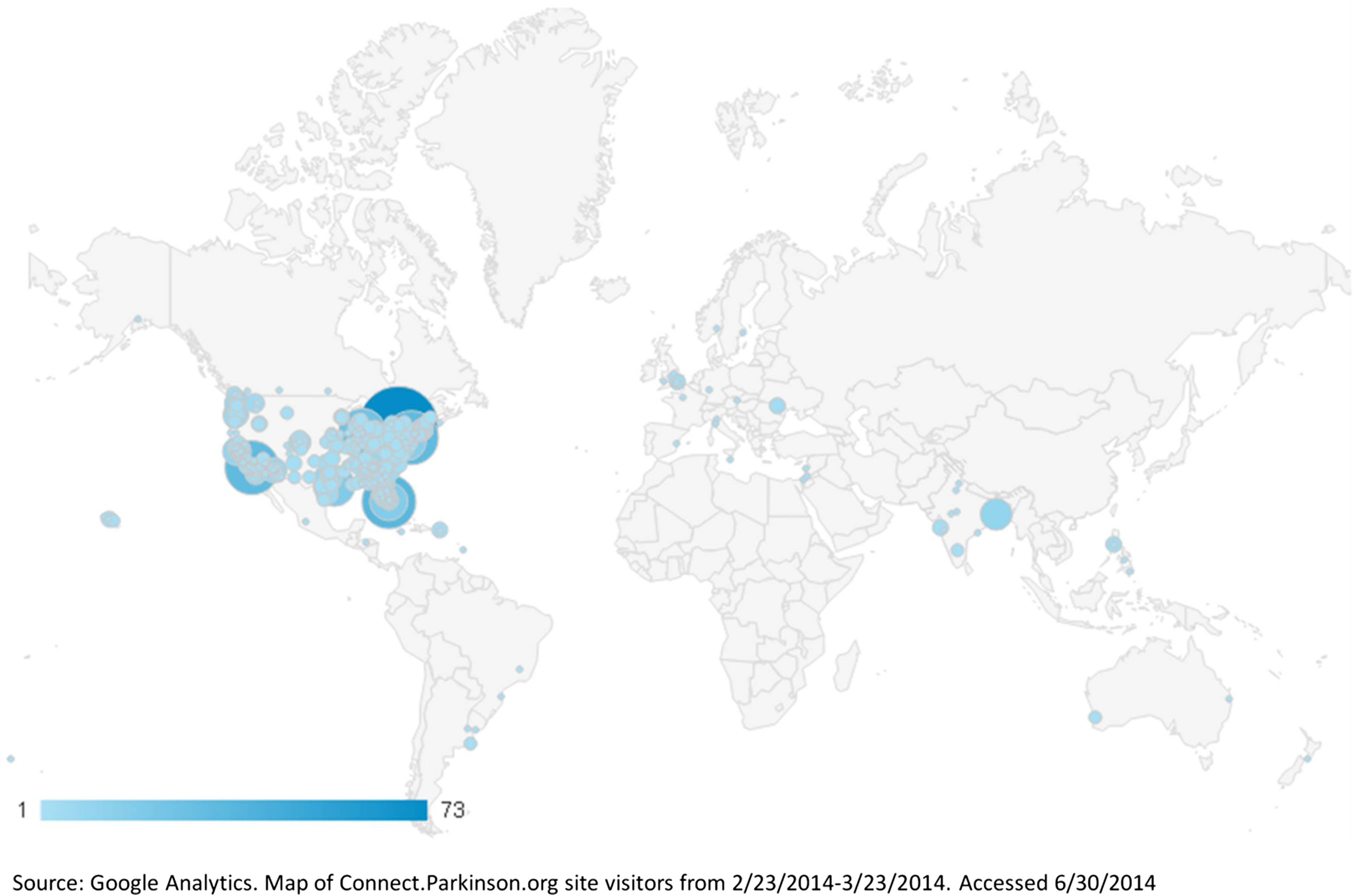

Supplement: Supplementary file 4 — Authors’ original file for figure 2 [file 13063_2014_2358_MOESM4_ESM.tif]

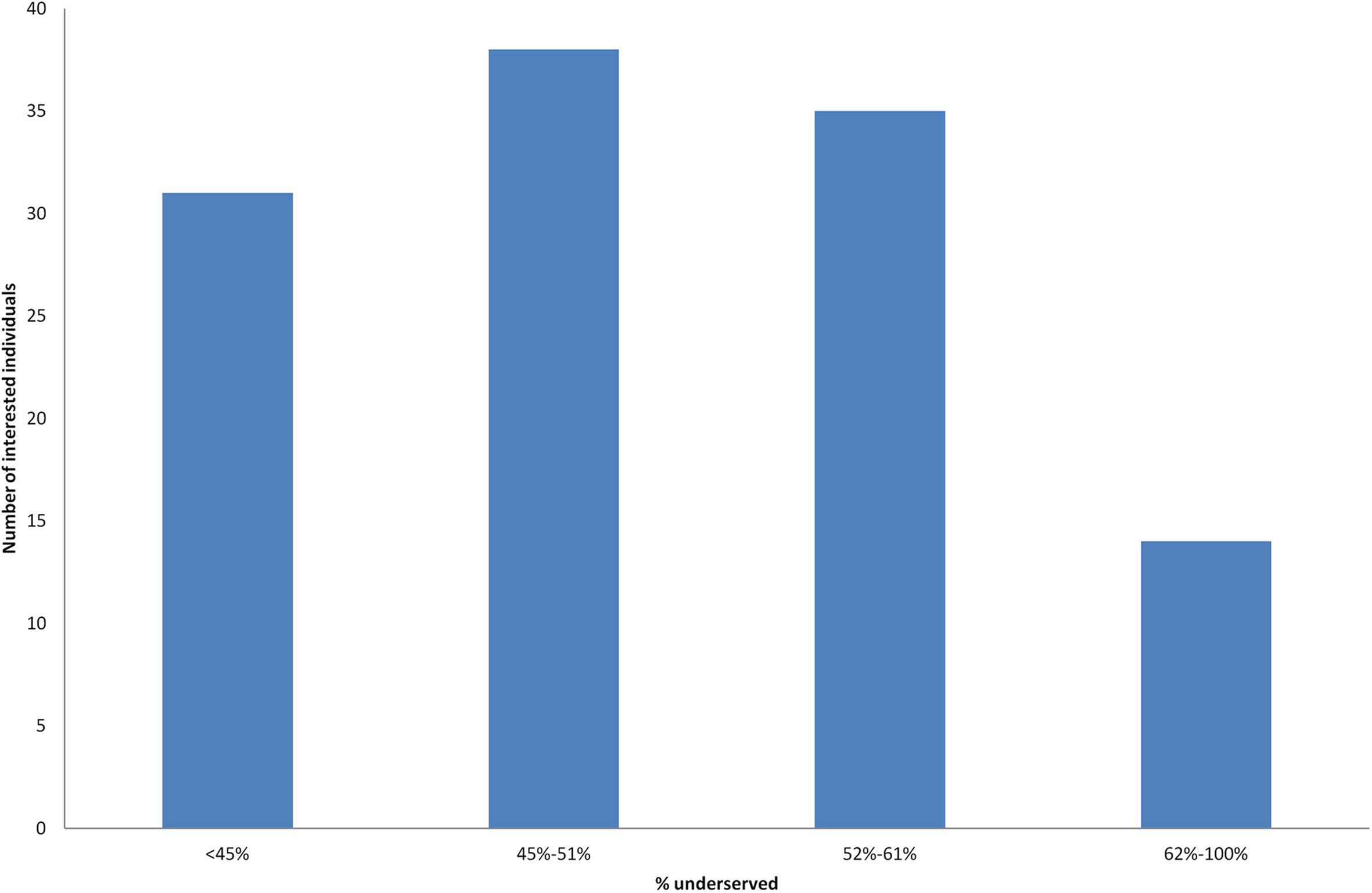

Supplement: Supplementary file 5 — Authors’ original file for figure 3 [file 13063_2014_2358_MOESM5_ESM.tiff]
